# Anal screening cytology

**DOI:** 10.1186/1742-6413-2-5

**Published:** 2005-02-16

**Authors:** Gladwyn Leiman

**Affiliations:** 1 University of Vermont, 111 Colchester Avenue, Burlington, VT 05446, USA

**Keywords:** Anal intraepithelial neoplasia, anal Pap, HIV-positive, anal cytology screening, ASIL cytomorphology, anoscopy

## Abstract

This issue of CytoJournal contains an article on screening for anal intraepithelial neoplasia in high-risk male patients. This accompanying Editorial focuses on current understanding of this relatively new disease entity, with insights as to the potential role of screening cytopathology in the epidemiology, pathophysiology and clinical management of this HIV and HPV related anal lesion, which predominates in male patients living long-term with AIDS. Mention is made of techniques of obtaining samples, methods of preparation, and morphologic classification. Issues of anoscopic confirmation, as well as topical and surgical management are emphasized. The similarity of initial experiences in anal screening to problems encountered early in cervical cancer screening programs several decades ago, are highlighted.

## Note

For corresponding research article please see Arian et al, 2005 [15]

## 

The subject of anal screening cytology has entered the epidemiologic and cytopathologic literature as a topic of interest over the last decade, and is highlighted in this issue of CytoJournal in an article by Arain and colleagues. Until recently, anal cancer was not considered to be a neoplasm of major public health concern [1]. It occurred infrequently, usually in older people, affecting women more often than men; further, being a neoplasm of low incidence, it remained under the radar in terms of screening potential. In some respects, anal cancer mirrored cervical cancer in unscreened women, presenting late in the course of the disease with a variety of pelvic symptoms, and having a protracted, ultimately fatal, course. Patients invariably became social outcasts, suffering from intolerable fecal complications, which presented major nursing challenges. Two aspects of this scenario have changed. First was the fairly recent introduction of effective modern treatment regimes for invasive anal squamous cancers utilizing chemo-radiation, leading to improvement in morbidity and long term survival [2,3]. The second significant alteration was seen in epidemiology, and this is the area which has come to involve screening cytopathology.

During the 1990s, in several European and North American cancer centers, an initially unaccountable increase in anal cancers was seen in younger people. It soon became apparent that HIV-positive homosexual males were affected in numbers greatly in excess of those expected. This was first noted in those urban areas in which large concentrations of homosexual men had been treated since the outbreak of the HIV epidemic. The term "males having sex with males" (MSM) was coined to cover these high-risk individuals. Preliminary information gleaned from screening programs in high HIV-positive incidence areas amongst MSM, showed detection rates of intraepithelial and early invasive neoplasia higher than any incidence ever recorded for cervical cancer screening. Further, HIV-negative homosexual MSMs, and HIV positive non-homosexual men (eg drug users) also exhibited an increased incidence of pre-malignant and invasive anal carcinomas. More recently, the syndrome of early onset anal cancer has been extended to include HIV-positive female patients, as well as females who are not HIV-positive, but who have genital HPV. All these groups show a higher incidence of abnormal anal cytopathology, though none quite as high as that found in HIV positive MSM. The unifying factor in most instances is ano-receptive intercourse, or extension of HPV infection from the genital tract to the anal mucosa, most obvious in the face of deficient immunity, or high viral load [4-6].

Epidemiologic studies have indicated that a prolonged preclinical phase precedes the onset of anal cancer in these high-risk groups. Just as in the cervix, there is a transitional zone (although not an abrupt squamocolumnar junction) in the anus. Rectal mucosa, with goblet cells, ends about one inch proximal to the external sphincter, giving way to a transitional epithelium, which in turn blends into a stratified squamous epithelium at the level of the anus. (External to the anus, any lesions which arise are considered to be primary skin lesions rather than anal lesions.) Almost all anal cancers develop in the transitional zone, where atypical, dysplastic and in-situ lesions are identifiable histologically. The cytologic counterparts are those of atypical cells of uncertain significance (ASCUS), low grade squamous intraepithelial lesions (LSIL) and high grade squamous intraepithelial lesions (HSIL). Once this natural history had been demonstrated and confirmed, it seemed natural that exfoliative cytology could be investigated as an "anal Pap smear" [7,8].

As with the cervix, the technique of obtaining the anal sample is critical to the success of screening. Standard colonic preparation is not required, but the rectum should be emptied prior to obtaining the anal sample. Brushes, brooms and Dacron swabs have all been used, the type of spatula probably being less important than the skill of the operator. The instrument is inserted to a depth of one and a half inches beyond the external sphincter, and subsequently withdrawn in a firm downward spiral movement incorporating 10–12 rotations, to ensure the device has made contact with the full surface area of the transformation zone. Some have used direct smears onto glass slides with immediate wet fixation; most centers, however, employ immediate insertion of the scraping device into liquid fixative for thin layer preparation. This appears both to improve adequacy and preservation, and also to eliminate any fecal contamination; residual material in the vial can be used for HPV studies if required, or for the creation of a bank of teaching slides.

There has been a relative dearth of literature on the cytomorphology of anal samples [9-11]. Classification according to Bethesda guidelines has been advised, implying similarity of the exfoliative cytology of atypical squamous cells of undetermined significance (ASC-US), anal intraepithelial lesions (A-SIL) and invasive squamous-cell neoplasms to those encountered in the cervix. This early in the development of the science of anal screening, and evident also in the current article in CytoJournal, there are indications that cytologic evaluation may not fully anticipate the severity of some lesions, when compared with biopsies taken simultaneously. Several groups have advised that all abnormal anal Paps should be followed by anoscopic evaluation, with biopsy confirmation when necessary. Anoscopy essentially mirrors colposcopy, enabling the viewer to see vascular abnormalities at magnification, and direct biopsies to the most abnormal-appearing areas. Despite these basic similarities, it is strongly stated in the literature that expertise in colposcopy does not equate to immediate competence in anoscopy; a significant learning curve exists for those wishing to acquire excellence in anoscopic technique, with associated accurate biopsy sampling. It is important that the anoscopist not be sidelined by visible condylomata, which may merely be sentinels of deeper flat lesions of higher grade. At the present time, lack of available expertise in this interventional follow-up of abnormal anal Paps may be the single limiting factor in any new screening program. It thus behooves those interpreting anal samples to be as proficient as they can be in pre-interventional assessment of the anal transformation zone.

Experience over the last decade suggests that anal cancer may be as highly appropriate a target for screening as is the cervix, in selected populations. The neoplasm is frequently encountered in well-defined high-risk groups. It has a detectable pre-malignant phase, and is amenable to easy cytologic sampling. Cytodiagnosis is reasonably sensitive and highly specific, and histologic confirmation is relatively easily obtained by well-trained personnel. If there is a current area of deficiency in such programs, it may well be in the treatment of intraepithelial lesions, which, as yet, has not been adequately assessed in large numbers of patients. It is known that highly active anti-retroviral therapy (HAART) does not appear to alter the pathophysiology of anal lesions once initiated. A variety of topical agents such as podophyllotoxin and imiquimod have been tried, as has intralesional interferon; superficial ablative therapies including liquid nitrogen, electrocautery, laser and LEEP have been attempted with varying success rates. Circumferential surgical resection almost inevitably results in unacceptable loss of sphincter control and soiling, but anoscopy-directed limited excision may prove less morbid. The fact that so many options are available implies perhaps that no single modality is yet considered sufficiently effective, with minimal complication [12,13]. This, too, is reminiscent of the early years of management of cervical pre-neoplasia, when a host of methods was pursued in attempted elimination of focal lesions of the squamocolumnar junction. As in the cervix, human ingenuity will undoubtedly prevail, and one or two forms of extirpation will emerge as both efficacious and uncomplicated.

An interesting consideration is whether or not anal cancer, and thereby anal cytopathology screening programs, would be of value in the developing world, particularly in Africa, India and China, where the bulk of the global incidence of HIV resides. This will depend on two very different factors. First is the nature of transmission. Unlike the situation in the developed world, AIDS in these regions is not essentially a disease of homosexual males; thus, without anal intercourse predominating, an upward trend in the incidence of anal cancer would seem unlikely. Anal screening programs would be unnecessary or cost-*in*effective in these communities. The second feature dictating the institution of screening programs in developing countries relates to antiretroviral therapy. Anal intraepithelial neoplasia and cancer are not encountered early in the progression of HIV/AIDS. Rather, they are late complications of patients living long-term with AIDS, usually implying patients living long-term on HAART [14]. Unless affordable very low cost antiretroviral drugs could be manufactured and distributed widely, it seems unlikely that patients in developing countries would survive into the time zone in which delayed neoplasms such as anal cancer become a public health priority.
